# Sensitivity, Specificity, and Accuracy of Ultrasound, Mammography, and ^18^F-FDG PET/CT in Diagnosing Breast Cancer Metastasis to Axillary Lymph Nodes: A Single-Center Experience

**DOI:** 10.3390/medicina62020320

**Published:** 2026-02-04

**Authors:** Gokmen Aktas, Hakan Buyukhatipoglu, Tulay Kus, Mehmet Emin Kalender, Hamit Yıldız, Talha Yıldız, Alper Sevinç, Seval Kul, Celaletdin Camci

**Affiliations:** 1 Division of Medical Oncology, Department of Internal Medicine, Gaziantep Medical Point Private Hospital, TR-27310 Gaziantep, Turkey; 2 Division of Medical Oncology, Department of Internal Medicine, Gaziantep State Hospital, TR-27470 Gaziantep, Turkey; 3 Gaziantep Oncology Hospital, Division of Medical Oncology, Department of Internal Medicine, School of Medicine, Gaziantep University, TR-27310 Gaziantep, Turkey; 4 Gaziantep University Hospital, Department of Internal Medicine, School of Medicine, Gaziantep University, TR-27310 Gaziantep, Turkey; 5 Department of Biostatistics, School of Medicine, Gaziantep University, TR-27310 Gaziantep, Turkey; 6 Department of Medical Oncology, Istanbul Tema Hospital, TR-34310 Istanbul, Turkey

**Keywords:** axillary lymph node metastasis, ultrasound, mammography, ^18^F-FDG PET/CT

## Abstract

*Background and Objectives*: Axillary lymph node (ALN) status is one of the most important prognostic factors in breast cancer. Numerous studies have evaluated less invasive methods for accurate staging. To investigate the diagnostic performance of ultrasound (US), mammography, and ^18^F-fluorodeoxyglucose positron emission tomography/computed tomography (^18^F-FDG PET/CT) in predicting axillary lymph node metastasis in breast cancer patients. *Materials and Methods*: Axillary involvement detected by US, mammography, and ^18^F-FDG PET/CT was analyzed in patients who underwent axillary dissection. Preoperatively, 365, 318, and 85 of 557 patients were evaluated with US, mammography, and PET/CT, respectively. The sensitivity, specificity, positive predictive value (PPV), negative predictive value (NPV), and accuracy of each imaging modality were calculated. *Results*: The sensitivity, specificity, PPV, NPV, and accuracy of US were 33.3%, 97.5%, 94.4%, 51.0%, and 57.8%, respectively. False-negative US findings were more frequent in T1 (84.6%) and N1 (76.3%) tumors (*p* < 0.001 for both). For mammography, these values were 12.9%, 97.74%, 88.89%, 44.67%, and 52.9%, while for ^18^F-FDG PET/CT they were 72.5%, 100%, 100%, 67.65%, and 82.5%, respectively. *Conclusions*: Ultrasound remains useful for evaluating axillary lymph node involvement in advanced breast cancer but is insufficient for early-stage disease.

## 1. Introduction

Assessment of axillary lymph node involvement remains a key prognostic determinant in patients with breast cancer. For many years, axillary lymph node dissection (ALND) was considered the gold standard for diagnosing axillary lymph node (ALN) metastases. However, due to the high morbidity associated with this procedure, numerous studies have investigated whether omission of ALND could be safe in selected low-risk patients. These studies suggested that occult axillary metastases may never become clinically evident, and that axillary dissection could be avoided in patients with small tumors and a clinically negative axilla [1,2,3].

Sentinel lymph node biopsy (SLNB) was later introduced as a less invasive approach for axillary staging in patients with clinically node-negative breast cancer. Although SLNB has a false-negative rate of approximately 10%, it provides comparable accuracy with markedly lower morbidity than ALND. Nevertheless, it remains an invasive procedure [4,5,6].

The Z0011 trial randomly assigned patients with clinically node-negative T1–T2 breast cancer who had one or two positive sentinel lymph nodes and were treated with breast-conserving therapy plus radiotherapy to receive either completion ALND or no further axillary surgery. The results demonstrated no statistically significant differences in local recurrence (*p* = 0.11), regional recurrence (*p* = 0.45), or overall survival between the two groups [7]. Similarly, a meta-analysis confirmed that axillary dissection may be safely omitted in early-stage breast cancer patients with limited sentinel node metastasis [8].

Although SLNB is less invasive than ALND, it still carries a risk of long-term morbidity [9]. Therefore, accurate non-invasive assessment of axillary status has gained importance to avoid unnecessary invasive procedures, including SLNB, particularly in low-risk patients.

In this context, accurate preoperative axillary staging has become increasingly important within contemporary treatment paradigms aimed at de-escalating axillary surgery. Reliable non-invasive imaging may play a key role in treatment planning by improving patient selection for surgical versus non-surgical axillary management. Enhanced imaging strategies have the potential to reduce overtreatment, minimize procedure-related morbidity, and support individualized decision-making, particularly in patients with early-stage disease and low nodal burden. Consequently, the diagnostic performance of commonly used imaging modalities warrants careful evaluation to clarify their contribution to modern, risk-adapted axillary management.

Ultrasound (US) is a simple and routinely used modality for preoperative axillary evaluation in many oncology centers. In selected clinical settings, imaging modalities such as magnetic resonance imaging (MRI) and ^18^F-fluorodeoxyglucose positron emission tomography/computed tomography (^18^F-FDG PET/CT) may also be incorporated into axillary assessment.

However, although up to 50% of patients with axillary involvement can be identified preoperatively by these imaging modalities, postoperative pathological evaluation often yields discordant results [10].

The aim of this study was to evaluate the diagnostic performance of ultrasound, mammography, and ^18^F-FDG PET/CT for the detection of axillary lymph node metastases in breast cancer based on sensitivity, specificity, positive and negative predictive values, and overall accuracy, as well as to examine associated clinicopathological factors.

## 2. Patients and Methods

### 2.1. Study Design

This retrospective, single-center diagnostic accuracy study was performed in compliance with the ethical principles outlined in the Declaration of Helsinki. The study protocol and informed consent procedures were reviewed and approved by the Independent Ethics Committee.

The primary aim of the study was to evaluate diagnostic performance metrics—including sensitivity, specificity, positive predictive value (PPV), and negative predictive value (NPV)—of ultrasound (US), mammography, and ^18^F-fluorodeoxyglucose positron emission tomography/computed tomography (^18^F-FDG PET/CT) for assessing axillary lymph node (ALN) status in surgically treated breast cancer patients.

### 2.2. Patient Selection

A total of 557 consecutive patients with primary invasive breast cancer who underwent axillary lymph node dissection (ALND) between January 2007 and June 2025 were included.

Eligibility required at least 10 dissected lymph nodes. Patients were excluded if they:

- Had stage IV disease;
- Had breast metastases from another primary tumor;
- Had received neoadjuvant chemotherapy before surgery.

Among these patients, 365 underwent preoperative axillary US, 318 underwent bilateral mammography, and 85 underwent preoperative ^18^F-FDG PET/CT.

Clinicopathological variables were retrieved from medical records, including TNM classification, tumor size, axillary lymph node status, histological grade, and the expression status of estrogen receptor (ER), progesterone receptor (PR), and HER2.

Preoperative imaging findings (US, mammography, and PET/CT) and postoperative pathological results were documented for each patient.

Diagnostic performance metrics (sensitivity, specificity, PPV, NPV, accuracy) were calculated and their associations with clinicopathologic features were analyzed.

### 2.3. Ultrasound (US)

US evaluation included assessment of size, morphology, presence of a hyperechoic hilum, and cortical thickness of axillary lymph nodes.

Nodes larger than 20 mm, with an absence of a fatty hilum, or showing abnormal cortical morphology were considered suspicious for metastasis.

All imaging examinations were interpreted by experienced radiologists specialized in breast imaging. Ultrasound and mammography studies were reviewed by radiologists with a minimum of five years of experience in oncologic breast imaging, while PET/CT images were interpreted by nuclear medicine physicians with comparable expertise. As this was a retrospective study, imaging interpretations were based on routine clinical reports generated at the time of examination. Formal assessment of interobserver variability was not performed; however, imaging interpretation followed standardized institutional criteria, and final pathological findings were used as the reference standard for diagnostic performance analysis.

### 2.4. Mammography

Mammograms were obtained for either screening or diagnostic purposes using dedicated mammography units.

### 2.5. ^18^F-FDG PET/CT

All patients fasted for at least 4 h before intravenous administration of ^18^F-FDG (dose: approximately 145 μCi/kg, maximum 20 mCi).

Blood glucose levels were confirmed to be below 200 mg/dL before injection. Imaging of the chest began 60 min (±15 min) after tracer injection.

The maximum standardized uptake value (SUV_max_) was used as the quantitative measure of FDG uptake and was calculated as:

$${\mathrm{SUV}}_{max}=\frac{\mathrm{maximum}\;\mathrm{activity}\;\mathrm{concentration}\;(\mathrm{MBq}/g)}{\mathrm{injected}\;\mathrm{dose}\;(\mathrm{MBq})/\mathrm{body}\;\mathrm{weight}\;(g)}$$

### 2.6. Statistical Analysis

The chi-square (χ^2^) test was used to evaluate associations between categorical variables, and the Z-test was used to compare proportions. For categorical variables, comparisons between subgroups were performed using the chi-square test. One category within each variable was defined as the reference group for statistical comparison.

Univariate analyses were chosen in accordance with the primary objective of the study, which was to evaluate and compare the diagnostic performance of imaging modalities rather than to identify independent predictors of false-negative results. Although multivariable modeling may provide additional insights into independent risk factors, it was not performed due to the retrospective design of the study, the heterogeneity of imaging subgroups, and the limited number of patients undergoing ^18^F-FDG PET/CT. The potential value of multivariable analysis is therefore acknowledged as a methodological consideration for future prospective studies. The effectiveness of diagnostic tests was assessed by calculating sensitivity, specificity, PPV, NPV, and 95% confidence intervals (CIs). Continuous variables were summarized using mean values with corresponding standard deviations (SDs), whereas categorical variables were presented as numbers and percentages. Statistical analyses were conducted using SPSS for Windows version 22.0 (IBM Corp., Armonk, NY, USA) and MedCalc version 15.0 (MedCalc Software, Ostend, Belgium). A *p*-value below 0.05 was considered to indicate statistical significance.

## 3. Results

This study included 557 consecutive patients with invasive breast cancer who underwent preoperative ultrasound (US), mammography, or ^18^F-FDG PET/CT between 2007 and 2025. The mean age was 49.6 ± 11.4 years. Histopathologically, 486 (92%) cases were invasive ductal carcinoma and 43 (8%) were invasive lobular carcinoma.

The clinicopathological characteristics of all patients are summarized in Table 1.

### 3.1. Ultrasound Analysis of Axillary Lymph Nodes

Among the 365 patients who underwent preoperative axillary US, 21.9% (n = 79) were reported to be positive for metastasis. Of these, 75 were true positives, 4 were false positives, 136 were true negatives, and 150 were false negatives.

Accordingly, the sensitivity, specificity, PPV, NPV, and accuracy of US for predicting axillary lymph node status were 33.3%, 97.5%, 94.4%, 51.0%, and 57.8%, respectively.

Analysis of the 150 false-negative cases according to clinicopathological features showed the following:

- False-negative rates based on tumor (T) stage were 84.6% (22/26) for T1, 67.2% (88/131) for T2, 59.5% (28/47) for T3, and 57.1% (12/21) for T4.

False-negative results were significantly more frequent for T1 tumors (*p* < 0.001).

- Based on nodal (N) stage, the false-negative rates were 76.3% (87/114) for N1, 63.1% (36/57) for N2, and 50.0% (27/54) for N3, with the highest rate in N1 disease (*p* < 0.001).

ER positivity was also associated with false-negative US findings (*p* = 0.021), while the correlations with PR status, HER2 status, and histologic grade were not statistically significant (Table 2 and Table 3).

### 3.2. Mammography Analysis of Axillary Lymph Nodes

Of the 318 patients who underwent preoperative mammography, 8.8% (n = 27) were reported to be positive for axillary metastasis. There were 24 true-positive, 3 false-positive, 161 false-negative, and 130 true-negative findings.

Accordingly, the sensitivity, specificity, PPV, NPV, and accuracy of mammography for axillary lymph node involvement were 12.9%, 97.74%, 88.89%, 44.67%, and 52.9%, respectively.

When US and mammography results were combined, sensitivity increased to 41.4%, whereas specificity decreased to 94.69%.

### 3.3. ^18^F-FDG PET/CT Analysis of Axillary Lymph Nodes

A total of 85 patients underwent preoperative ^18^F-FDG PET/CT for axillary evaluation. Among them, 38.8% (n = 33) were reported as positive for axillary metastasis. All positive PET/CT results were true positives, and no false-positive results were observed. There were 16 false-negative and 36 true-negative cases.

Thus, the sensitivity, specificity, PPV, NPV, and accuracy of PET/CT were 67.3%, 100%, 100%, 69.2%, and 81.1%, respectively. Analysis of the 52 negative PET/CT results showed that among the 16 false-negative cases, 10 (62.5%) were N1, 3 (18.8%) were N2, and 3 (18.8%) were N3, indicating a significant association between lower nodal stage and higher false negativity (*p* = 0.013). Tumor size, ER status, PR status, HER2 expression, and histologic grade were not significantly associated with false-negative PET/CT results.

## 4. Discussion

Accurate clinical staging of breast cancer is crucial for determining the extent of surgery and for planning adjuvant or neoadjuvant therapy. In this context, preoperative axillary imaging plays a key role in disease staging. Reliable axillary evaluation can prevent unnecessary radical axillary dissection and even sentinel lymph node biopsy (SLNB), which, although less invasive, remains a surgical procedure with potential morbidity. In this retrospective study, we evaluated the diagnostic performance of ultrasound (US), mammography, and ^18^F-FDG PET/CT in detecting axillary lymph node (ALN) metastases in breast cancer and examined the relationship between imaging findings and clinicopathological factors in a Turkish patient cohort.

US evaluation included nodal size, morphology, presence of a hyperechoic hilum, and cortical thickness [11]. Lee et al. reported that the overall PPV and NPV of US for detecting metastatic nodal involvement were 0.81 and 0.60, respectively, with a sensitivity of 53.7%, specificity of 85.1%, and accuracy of 67.9%. The US features most strongly associated with malignancy were the absence of a hyperechoic hilum (*p* = 0.003) and an increased cortical thickness (*p* = 0.03). Patients with a metastatic nodal burden ≥ 20% were more likely to have abnormal findings on axillary US (*p* = 0.009) [12]. Previous studies have consistently shown that axillary US has a low negative predictive value, and negative results cannot reliably exclude nodal metastases. Preoperative axillary US alone is not sufficiently sensitive or specific enough to replace SLNB because of the substantial number of false-negative findings in patients with invasive breast cancer, although it can effectively identify most cases of N2 or N3 disease [13]. Thus, US remains particularly useful for detecting advanced axillary disease and avoiding underestimation before SLNB.

Schipper et al. evaluated clinically T1–T2N0 breast cancer patients treated with breast-conserving therapy and reported an overall NPV of 97.7%, but only 50% among those who were clinically N0 and pathologically N2–3 [14]. Similarly, Choi et al. found an NPV of 80.8%, with 15.5% of patients having N1 disease and 3.7% having N2–3 disease despite negative preoperative US findings. False-negative rates decreased as T stage increased (*p* < 0.05) [15]. Although a negative axillary US generally excludes the presence of pN2–pN3 disease, it cannot accurately distinguish between pN1 and pN2–pN3 involvement. Yan-Na Zhang et al. reported an overall false-negative rate of 30.6% and a false-positive rate of 18.2% for US. The false-negative rates for pathologic N1, N2, and N3 disease were 46.2%, 21.8%, and 9.3%, respectively. In patients with stage T1 disease and fewer than three metastatic lymph nodes, the false-negative rate reached 52.2%, which correlated with larger tumor size (*p* < 0.001), higher tumor grade (*p* = 0.009), and ER/PR expression (*p* = 0.009), but not with HER2 or Ki-67 status [16]. Similarly, Park et al. reported a 42.4% false-negative rate in both US and US-guided fine-needle aspiration (US-FNA) of the ALN, which was significantly associated with positive ER (*p* = 0.003), positive PR (*p* = 0.001), higher T stage (*p* = 0.004), and lymphovascular invasion (*p* = 0.001) [17]. In contrast, Johnson et al. reported no significant association between false-negative US results and tumor grade or ER/PR status [18].

Meretoja et al. retrospectively evaluated patients with negative axillary US findings and found that 30.5% had ALN metastases detected by SLNB or ALND. Factors associated with nodal metastases included younger age (*p* = 0.001), larger tumor size (*p* = 0.001), multifocality (*p* = 0.001), lymphovascular invasion (*p* = 0.001), higher histologic grade (*p* = 0.001), molecular subtype (*p* = 0.001), and palpable tumor (*p* = 0.001), whereas the associations with ER (*p* = 0.24), PR (*p* = 0.18), and HER2 (*p* = 0.22) status were not significant [19]. Although axillary US may not be as accurate as SLNB in predicting nodal involvement, it can still assist in planning adjuvant chemotherapy and/or radiotherapy in patients who do not undergo axillary surgery, thereby guiding treatment decisions when lymph node status is unknown.

Xuemei Zhang et al. retrospectively evaluated the diagnostic accuracy of ^18^F-FDG PET/CT for detecting axillary, internal mammary, and supraclavicular lymph node metastases in clinically node-negative breast cancer patients. Using a SUV_max_ cut-off of 2.0, the sensitivity, specificity, overall accuracy, PPV, and NPV were 46.3%, 91.1%, 79.8%, 63.3%, and 83.6%, respectively. The false-negative and false-positive rates were 54% and 9%, respectively. PET/CT was useful in identifying distant metastases but had limited value for regional lymph node evaluation [20]. Similarly, Riegger et al. compared PET/CT with US and reported that PET/CT demonstrated sensitivity, specificity, PPV, NPV, and accuracy rates of 54%, 89%, 77%, 74%, and 75%, respectively, whereas US showed rates of 38%, 78%, 54%, 65%, and 62%. PET/CT was significantly more accurate than US (*p* = 0.019), although the difference in sensitivity was not statistically significant (*p* = 0.058) [21]. Another study also found that PET/CT was not superior to US in axillary staging but was more effective in detecting distant metastases. The diagnostic accuracy of PET/CT correlated with ALN size and the SUV_max_ of the primary tumor (*p* = 0.02 and *p* = 0.04) [22].

Recent large-scale studies and meta-analyses have provided further insight into the evolving role of ^18^F-FDG PET/CT in axillary staging of breast cancer. Several systematic reviews and meta-analyses have demonstrated that, while PET/CT generally exhibits high specificity, its sensitivity for axillary lymph node metastasis remains modest, particularly in early-stage disease, limiting its utility as a standalone staging modality [23]. For example, meta-analytic data indicate pooled sensitivity values of around 50–60% for PET/CT in lymph node metastasis detection, with a specificity consistently above 80% in larger pooled cohorts [24]. Moreover, comparative analyses incorporating hybrid PET/MRI suggest that integrated imaging approaches may yield higher diagnostic performance than PET/CT alone, particularly when distinguishing metastatic involvement in complex nodal basins [24]. Nonetheless, the diagnostic value of PET/CT appears to be influenced by tumor burden, stage, and imaging protocols, and it is most informative in patients with a higher clinical stage or greater nodal burden [25]. These findings align with our results, reinforcing the notion that ^18^F-FDG PET/CT, while valuable in selected clinical scenarios, currently cannot supplant histopathological staging for routine detection of axillary metastases.

In the present study, the sensitivity and NPV of US were lower than those reported in the literature, whereas their specificity and PPV were higher. This discrepancy may reflect a tendency among Turkish radiologists to be more conservative in interpreting suspicious axillary findings as malignant. In our subgroup analyses, false-negative results correlated significantly with nodal stage and tumor size, consistent with previous studies. Except for ER status, other clinicopathologic factors such as PR status, HER2 status, and histologic grade were not significantly associated with false negativity, which is also in agreement with earlier reports. Although the number of patients who underwent ^18^F-FDG PET/CT was limited, both the sensitivity and specificity were higher than in most published studies. Consistent with earlier reports, false-negative results were more common in early-stage nodal disease, suggesting that PET/CT remains less reliable for detecting micrometastatic axillary involvement.

The combined use of ultrasound and mammography resulted in a higher sensitivity (41.4%) compared with either imaging modality alone, underscoring the complementary nature of these techniques in preoperative axillary evaluation. Although this level of sensitivity remains insufficient to replace invasive staging procedures such as sentinel lymph node biopsy, the observed improvement suggests that combining modalities may mitigate some of the inherent limitations associated with single-technique assessments. In particular, this approach may reduce underestimation of axillary involvement by enabling the identification of radiologically suspicious lymph nodes that could otherwise be overlooked when ultrasound or mammography is used in isolation.

From a clinical standpoint, the combined application of ultrasound and mammography may function as an initial, non-invasive screening strategy that enhances risk stratification prior to surgical decision-making. By improving the detection of patients with a higher likelihood of nodal metastasis, this strategy may assist clinicians in selecting patients who would benefit most from further diagnostic workup or more aggressive axillary evaluation. Conversely, in patients with consistently negative findings across both modalities, combined imaging may provide additional reassurance while still acknowledging the necessity of histopathological confirmation in early-stage disease.

This study has several important limitations that warrant careful consideration when interpreting the results. First, the retrospective, single-center design introduces inherent selection bias and restricts the generalizability of the findings to broader clinical settings. Differences in institutional imaging protocols, diagnostic thresholds, and radiologist expertise may substantially influence diagnostic performance, and these factors cannot be fully accounted for in a retrospective analysis.

Second, there was marked heterogeneity in the number of patients evaluated with each imaging modality. While ultrasound and mammography were performed in the majority of the cohort, only a relatively small subset of patients underwent ^18^F-FDG PET/CT. This imbalance limits the statistical power and increases the risk of overestimating diagnostic performance, particularly in light of the absence of false-positive PET/CT findings. Such perfect specificity is uncommon in routine clinical practice and is more likely attributable to patient selection and referral patterns rather than intrinsic diagnostic superiority.

Another important limitation is the extended study period, spanning nearly two decades. During this time, significant advancements occurred in ultrasound equipment, PET/CT scanner resolution, image reconstruction techniques, diagnostic criteria, and breast cancer treatment guidelines. These temporal changes may have influenced imaging accuracy and clinical decision-making. However, due to the retrospective nature of the dataset and incomplete temporal stratification, era-specific analyses could not be reliably performed.

Furthermore, the statistical analysis was limited to univariate comparisons. Given that tumor size, nodal stage, hormone receptor status, and histological grade are biologically and clinically interrelated, the absence of multivariable modeling restricts the ability to identify independent predictors of false-negative imaging results. Finally, interobserver variability and blinding of imaging interpretation were not evaluated, which represents a relevant limitation in retrospective imaging studies and may have influenced diagnostic consistency.

## 5. Conclusions

In conclusion, ultrasound (US) is useful for assessing axillary lymph node involvement in advanced breast cancer, but its diagnostic performance is limited in early-stage disease. Combining US with mammography modestly increases sensitivity, while ^18^F-FDG PET/CT remains of limited value in early nodal stages. These findings support the ongoing de-escalation of axillary surgery in appropriately selected patients.

## Figures and Tables

**Table 1:** Clinicopathological characteristics of patients.

Variable	Number (%) of Cases
**Age**	
<65	485 (87.1)
≥65	72 (12.9)
**Tumor Size**	
T1 (<2 cm)	66 (11.8)
T2 (2–5 cm)	362 (64.9)
T3 (>5 cm)	91 (16.3)
T4 *	38 (6.8)
**Axillary Lymph Node Metastasis**	
N0	216 (38.7)
N1	174 (31.2)
N2	89 (16)
N3	78 (14)
**ER expression**	
Positive	401 (72)
Negative	165 (28)
**PR expression**	
Positive	385 (69.1)
Negative	172 (30.9)
**HER-2 Expression**	
Positive	161 (28.9)
Negative	296 (71.1)
**Histological Grade**	
I	42 (7.6)
II	246 (44.2)
III	268 (48.2)

* Tumor has spread into chest wall or skin, or is an inflammatory carcinoma.

**Table 2:** Characteristics of 150 patients with false-negative results on axillary US.

Clinicopathological Variable	False Negative/All Negative (%)	*p* Value
**Tumor Size**		
T1 (<2 cm)	22/26 (84.6)	0.029
T2 (2–5 cm)	88/131 (67.2)	0.31
T3 (>5 cm)	28/47 (59.5)	0.84
T4 *	12/21 (57.1)	Reference ^1^
**Axillary Lymph Node Metastasis**		
N1	87/114 (76.3)	<0.001
N2	36/57 (63.1)	0.165
N3	27/54 (50)	Reference ^1^
**ER expression**		
Positive	119/167 (71.2)	0.021
Negative	28/51 (54.9)	
**PR expression**		
Positive	116/162 (69.4)	0.055
Negative	31/56 (55.3)	
**HER-2 Expression**		
Low (Negative)	39/63 (61.9)	0.27
High (Positive)	108/155 (69.6)	
**Histological Grade**		
I	5/5 (100)	0.088
II	67/90 (74.4)	0.069
III	77/123 (62.6)	Reference ^1^

* Tumor has spread into chest wall or skin, or is an inflammatory carcinoma. ^1^ Categories marked as “Reference” represent the baseline groups for statistical comparison.

**Table 3:** Characteristics of 52 patients with negative results on ^18^FDG PET\CT.

Clinicopathological Variable	False Negative/All Negative (%)	*p* Value
**Tumor Size**		
T1 (<2 cm)	1/8 (12.5)	0.600
T2 (2–5 cm)	10/32 (31.2)	0.802
T3 (>5 cm)	4/8 (50)	0.427
T4 *	1/4 (25)	Reference ^1^
**Axillary Lymph Node Metastasis**		
N1	10/16 (62.5)	0.013
N2	3/16 (18.8)	1.00
N3	3/16 (18.8)	Reference ^1^
**ER expression**		
Positive	13/41 (31.7)	0.781
Negative	3/11 (27.3)	
**PR expression**		
Positive	13/33 (39.4)	0.078
Negative	3/19 (15.8)	
**HER-2 Expression**		
Low (Negative)	4/9 (44.4)	0.27
High (Positive)	12/43 (27.9)	0.33
**Histological Grade**		
I	1/2 (50)	0.40
II	10/28 (35.7)	0.324
III	5/22 (22.7)	Reference ^1^

* Tumor has spread into chest wall or skin, or is an inflammatory carcinoma. ^1^ Categories marked as “Reference” represent the baseline groups for statistical comparison.

## Data Availability

The data presented in this study are available from the corresponding author upon reasonable request. The data are not publicly available due to ethical and privacy restrictions related to patient information.
